# Molecular effects of polystyrene nanoplastics on human neural stem cells

**DOI:** 10.1371/journal.pone.0295816

**Published:** 2024-01-03

**Authors:** Raquel Martin-Folgar, Mª Carmen González-Caballero, Mónica Torres-Ruiz, Ana I. Cañas-Portilla, Mercedes de Alba González, Isabel Liste, Mónica Morales

**Affiliations:** 1 Grupo de Biología y Toxicología Ambiental, Departamento de Física Matemática y de Fluidos, Facultad de Ciencias, UNED. Urbanización Monte Rozas, Las Rozas (Madrid), Spain; 2 Environmental Toxicology Unit, Centro Nacional de Sanidad Ambiental (CNSA), Instituto de Salud Carlos III (ISCIII), Majadahonda (Madrid), Spain; Xiangtan University, CHINA

## Abstract

Nanoplastics (NPs) have been found in many ecological environments (aquatic, terrestrial, air). Currently, there is great concern about the exposition and impact on animal health, including humans, because of the effects of ingestion and accumulation of these nanomaterials (NMs) in aquatic organisms and their incorporation into the food chain. NPs´ mechanisms of action on humans are currently unknown. In this study, we evaluated the altered molecular mechanisms on human neural stem cell line (hNS1) after 4 days of exposure to 30 nm polystyrene (PS) NPs (0.5, 2.5 and 10 μg/mL). Our results showed that NPs can induce oxidative stress, cellular stress, DNA damage, alterations in inflammatory response, and apoptosis, which could lead to tissue damage and neurodevelopmental diseases.

## 1. Introduction

Large plastic production [1, 2] and use have resulted in the release of plastic waste into aquatic, terrestrial and even aerial ecosystems, being a great problem to current and future generations [3]. These plastic materials with time, UV radiation, environmental variables, etc. can fragment into small micro (1 μm—5 mm, microplastics, MPs) and nano (< 1 μm, nanoplastics, NPs) sized particles [4, 5]. MPs and NPs are made of different plastic types such as polypropylene (PP), polyethylene (PE), or polystyrene (PS) [6, 7]. NPs and MPs are emerging pollutants which can accumulate in organisms and whose toxic and health effects have made them one of the international environmental, public health, and animal health priority targets [7, 8]. The MPs and NPs can enter the human body by inhalation, ingestion, and skin contact [9]. However, the answer to how these NMs pass through the gut, lungs and epithelia to other organs is very scarce. There is scientific evidence that they can reach the systemic circulation, penetrate, and accumulate in different tissues and organs such as brain, eyes, spleen, liver, bone marrow, etc. [9–11]. Other studies have shown that MPs and NPs produce impacts on development, growth, reproduction, behavior, and mortality in aquatic [12] and terrestrial animals [13]. In addition, some research suggests that NPs can accumulate in living organisms and can cause inflammation [14], oxidative stress [7], dysregulation of energy metabolism [15], endocrine disruption [16], apoptosis [17], and growth inhibition [18], among others. It has been suggested that oxidative stress can cause cellular damage that can lead to neuronal disorders, as they produce inhibition of acetylcholinesterase (AChE) activity and alter neurotransmitter levels [19, 20]. In addition, studies from our group, demonstrate that PS NPs cause increased gene expression of oxidative stress markers, apoptosis, inflammation, and inhibition of neurotransmitter (*AChE*) gene expression in zebrafish embryos (Zfe) [21]. Furthermore, these particles are able to enter the Zfe central nervous system (CNS), and cause deleterious effects, as well as endocrine system effects and behavioral alterations [11]. Their potential neurodevelopmental toxicity is a concern, as these nanomaterials can cross the Blood-Brain Barrier (BBB) [22]. In aquatic organisms the presence of latex NPs and PS NPs has been observed in various organs, including the brain of Medaka *(Oryzias latipes)* and other fish [23], producing behavioral disorders [24]. Currently, there are very few studies investigating the potential neurotoxicity of PS NPs in neural cell models. However, PS NPs have shown to reduce viability, activate inflammatory response, and induce apoptosis in other types of human cells such as A549 alveolar cells [25]. The relationship between NPs and alterations at the level of gene expression in stem cells is so far unknown [26].

The development of *in vitro* models for toxicity testing is currently being encouraged [27]. These models better reproduce human physiology and are replacing animal models. The main trend is to use immortalized cell lines derived from cancer tissues and, recently, models that include stem cells have also begun to be used [28]. These new models have advantages over cancer lines in that they do not have an altered genotype, which allows the presence of physiologically more important cell types and makes it possible to estimate interindividual variation [29, 30]. In our work, we analyzed the effects of NPs in an *in vitro* model based on human neural stem cells (hNS1), in order to obtain a real response of the effects caused by exposure to nanoparticles and to obtain more reliable results. To address this work, gene expression of different metabolic pathways, such as stress response (*hsp27/hspB1*, *hsp60*, *hsp70/hspA5*, *and hsp90α*), DNA repair (*xrcc1*, *gad45a*, *rad51*), oxidative damage response (*Cu/ZnSOD 1*, *MnSOD 2*), apoptotic response (*Cas3a*, *Cas7*, *p53*, *Bcl2*), and mitochondrial response (*Cox5A*), were analyzed after PS NP exposure, as biomarkers of NPs damage. Cells were exposed to concentrations similar to those present in the environment and/or used in previous studies (0.5, 2.5, and 10 μg/mL) for 4 days [11, 21, 31–34]. Currently, there is limited information on the neurotoxicity of NPs in mammals [22] and there are no data on the concentration and bioaccumulation of these nanomaterials in humans. This study provides a platform to examine the impacts of Polystyrene nanoparticles (PS NPs) on human neural stem cells, focusing on potential damage at the neurotoxicogenomic level in humans as the mechanisms of toxicity are largely unknown.

## 2. Material and methods

### 2.1 Cell culture

Cell line hNS1 was used as a model of human neural stem cells (hNSCs) that has been previously characterized [35–38]. This cell line is non-transformed, derived from human fetal forebrain and immortalized with v-myc [39]. Cells were cultured on poly-L-lysine (10 μg/mL; Sigma) coated plastic plates and proliferated in human stem cell (HSC) medium [Dulbecco’s Modified Eagle Medium (DMEM)/F12 with GlutaMAX-I medium (Gibco) containing 0.26% AlbumaMAXb (Gibco), 0.6% glucose (Merck), N2 Supplement 1X (Gibco), 5 mM HEPES (Gibco), penicillin/ streptomycin 1× (P/S; Lonza), non-essential aminoacids 1X (Gibco)] and supplemented with 20 ng/mL epidermal growth factor (EGF; PreproTech) and 20 ng/mL basic fibroblast growth factor (FGF2; PreproTech). hNS1 cells were differentiated by withdrawal of growth factors (EGF and FGF2) and addition of 0.5% heat-inactivated fetal bovine serum (FBS) (differentiation medium). Cells were kept in an incubator set to 37 °C and 5% CO_2_.

### 2.2 Nanoparticle preparation and cells exposure

Pristine PS particles with an average diameter of 30 nm were purchased from Thermo ScientificTM (Spain). NP of 1.05 g/cm³ density were provided as a 1% and 10% solution in water respectively, both with < 2% surfactant (SDS) to prevent agglomeration, and < 0.05% the antibacterial agent NaN_3_ (only fluorescently labelled particles). Nanoplastic particle size and charge were characterized in cell culture medium by nanoparticle tracking analysis (NTA), direct light scattering (DLS), and electron microscopy [21]. We published this characterization previously [21]. All cells were treated for 4 days with PS NPs after 2 days of seeding in the culture plate [3x10^5^ cells/well (P6)]. The stock solution was shaken and sonicated for 10 min before utilization. The NPs were diluted in differentiation medium at 0.5, 2.5, and 10 μg/mL. The cells were differentiated for 4 days under exposure to PS NPs because preliminary studies showed that during that time of cell differentiation, a higher amount of NPs was found inside the treated cells. The stock solution of NPs was vortexed and sonicated for 10 min before use. NPs were diluted in differentiation medium to a final concentration of 0.5, 2.5 and 10 μg/mL. These concentrations were selected based on literature published. Due to methodological difficulties, very little work had been done measuring nanoparticles in the environment. However, a recent article by Materić [34] using novel methodology to measure NPs, has found these particles in Swedish lakes and streams with an average concentration of 0.56 mg/L. It is only logical to assume that concentrations in more populated/contaminated areas will be higher.

### 2.3 RNA extraction and cDNA synthesis

Total RNA extraction was performed from 0.5, 2.5, and 10 μg/mL treated 6x10^5^ hNS1 cells and untreated control cells using a commercial kit (Trizol, Invitrogen) according to the manufacturer’s protocol. Cells frozen at -80 °C were homogenized in 500 μL of Trizol and left for 5 min at room temperature. Next, 0.2 volumes of chloroform were added to each sample, mixed, and left for 5 minutes at room temperature. Samples were centrifuged at 15,000 g for 15 minutes at 4 °C. The aqueous phase containing RNA was transferred to an eppendorf tube and precipitated with isopropyl alcohol (0.5 v/v), washed with 70% ethanol, and resuspended in DEPC water. Subsequently, RNA was treated with RNase-free DNase (Roche) followed by phenolization (phenol: chloroform: isoamyl alcohol-extracted, 25:24:1, and isopropanol-precipitated), and resuspended in DEPC water [40]. The quality and quantity of total RNA was determined by agarose electrophoresis and absorbance (Biophotomer Eppendorf). RNA was stored at -80 °C until use. The cDNA was synthesized from 500 ng total RNA, 500 ng Oligo dT20 (Invitrogen), and 100 u/μL MMLV enzyme (Invitrogen, Germany). The cDNA was frozen at -20 °C.

### 2.4 Real-time PCR

cDNA was used as a template in real-time PCR to analyse the messenger RNA (mRNA) expression profile of genes related to cell stress (*hsp27/hspB1*, *hsp90α*, and *hsp70/hspA5*), oxidative stress (*Cu/ZnSOD 1*, *MnSOD 2*, and *cat*), DNA repair (*gadd45α*, *rad51*, and *xrcc1*), apoptosis (*Cas3a*, *Cas7*, *Bcl2*, and *p53*), inflammatory (*iL-6* and *iL-8*), and mitochondrial (*cox 5A*) responses. To amplify the sequence of the genes analysed in this study, oligonucleotides were designed from the GenBank accession sequences (Table 1). Treated cells RNAs were compared with RNAs extracted from control cells. The reaction was performed under the following conditions: initial denaturation at 95 °C for 3 min and 40 cycles of denaturation at 95 °C for 5 s; annealing at 58 °C for 15 s; and elongation at 65 °C for 10 s. The sequences of the oligonucleotides designed in this study for each of the mentioned genes are shown in Table 1. All samples were analysed in duplicate, and two replicates of each plate were performed. *ADH*:*ubiquinone oxidoreductase subunit B4* (*NDUFB4*) and *Ribosomal protein S27* (*RPS27*) genes, with a coefficient of variation < 0.25 and an M-value < 0.5, were used as endogenous reference controls to normalize the expression data of the selected study genes. PCR efficiency was performed by making calibration curves. A standard curve based on five dilutions of an equimolar mixture of cDNA samples was produced in triplicate to verify the amplification efficiency of each gene (Table 1).

**Table 1:** Oligonucleotide sequences.

Gene name	Function	Gene symbol	Accession number	Primer (5’–3’)	RT-PCR product size (bp)
**NADH: ubiquinone oxidoreductase subunit B4**	Reference gene	*NDUFB4*	NM_004547.6	F: TTGGATCGAACATTTCACCTCTCA R1: GTCTGCTTCTGTGTTGTTAGGG	176
**Ribosomal protein S27**	Reference gene	*RPS27*	NM_001030.6	F: CGAGAACATGCCTCTCGCAAAG R: AGCATCCTGGGCATTTCACAT	128
**Cytochrome c oxidase subnit 5A mitochondrial**	Mitochondrialresponse	*Cox5A*	NM_004255.4	F: GGCTTAGGGGACTGGTTGTC R: CCGTAAGAGGGCTTGGCTAC	133
**Superoxide dismutase 1**	Antioxidant activity	*Cu/ZnSOD1*	NM_000454.5	F: ATGACTTGGGCAAAGGTGGA R: GGGCCTCAGACTACATCCAAG	120
**Superoxide dismutase 2**	Antioxidant activity	*MnSOD2*	NM_001024465.3	F: GCACTAGCAGCATGTTGAGC R: TTGATGTGAGGTTCCAGGGC	139
**Catalase**	Antioxidant activity	*cat*	NM_001752	F: CTGACTACGGGAGCCACATC R: GATGAGCGGGTTACACGGAT	184
**Heat shock protein**	Stress response	*hsp90α*	NM_001017963.3	F: GTGCTCGAGTCACATTCTGC R: CAACCCTTGGAGCAGCTAGT	174
**Heat shock protein**	Stress response	*hsp70/hspA5*	NM_005347	F: TGAACCCTAGCTGTGTCAGA R: GCACCAGCCTGTCCTTTATT	222
**Heat shock protein**	Stress response	*hsp27/HspB1*	NM_001540.5	F: GGCCCAGAAGCTGCAAAATC R: AAAGAACACACAGGTGGCGG	112
**Growth arrest and DNA-damage-inducible**	DNA repair	*gadd45*α	NM_001924.4	F: CACTGTCGGGGTGTACGAAG R: GTTGATGTCGTTCTCGCAGC	157
**X-ray repair cross complementing 1**	DNA repair	*xrcc1*	NM_006297.3	F: CCGATACGTCACAGCCTTCA R: TGTAGATCCATCGGGGACGA	152
**DNA repair protein RAD51 homolog 1**	DNA repair	*rad51*	NM_002875.5	F: GCAGTGCAAGCTATTTCAAGACA R: GCACAATCATCTGCAAGTGGG	123
**Interleukin**	Inflammatory response	*IL-6*	NM_000600.5	F: TGCAATAACCACCCCTGACC R: GTGCCCATGCTACATTTGCC	98
**Interleukin**	Inflammatory response	*IL-8*	BC013615.1	F3: GGCAACCCTAGTCTGCTAGC R3: TAAAGTGCTTCCACATGTCCTC	139
**Tumor necrosis factor alpha**	Inflammatory response	*TNFα*	NM_000594.4	F: AGAACTCACTGGGGCCTACA R: GCTCCGTGTCTCAAGGAAGT	177
**Tumor suppression**	Inflammatory response	*p53*	NM_000546.6	F: TTCTGTCCCTTCCCAGAAAACC R: AACCCACAGCTGCACAGGGC	156
**Apoptosis regulator Bcl-2**	Apoptotic response	*Bcl2*	NM_000633.3	F: CCTATCTGGGCCACAAGTGAA R: ACAGCCTGCAGCTTTGTTTC	122
**Caspase-3**	Apoptotic response	*Cas3a*	NM_004346.4	F: TGGTTTGAGCCTGAGCAGAG R: TGGCAGCATCATCCACACAT	122
**Caspase-7**	Apoptotic response	*Cas7*	NM_001227.5	F: TGGTTTGAGCCTGAGCAGAG R: TGGCAGCATCATCCACACAT	142

Amplification of a single DNA fragment was confirmed by analysing the melting curve after amplification. All samples were analysed in duplicate and three independent PCR replicates were performed for each experiment. Cycle threshold (Ct) values were converted to relative gene expression levels by using the 2-DDCt method and Bio- Rad CFX Manager 3.1 software.

### 2.5 Statistical analysis

The RNA levels obtained were normalized against *NADH*: *ubiquinone oxidoreductase subunit B4* (*NDUFB4*) and *Ribosomal protein S27* (*RPS27*) reference genes. The comparison between the control and the treated larvae were done using the variance analysis test (ANOVA) with Dunnett’s multiple comparison tests. A level of significance is indicated: p≤0.05 (*). All statistical tests were performed with SPSS^®^ 27.0 (SPSS Incorporated, Chicago IL, USA).

## 3. Results and discussion

Electron microscopy characterization of PS NPs showed that these particles were round with an average size of 25.1 ± 4 nm [21]. However, nanoparticle tracking analysis (NTA) analysis in cell culture medium showed agglomeration of these particles, even after sonicating. Particle size distribution and charge was as follows: 112.4 ± 37.5 nm size and -25 ± 1.06 mV charge for the 0.5 μg/mL concentration; 112.0 ± 44.2 nm and -33.8 ± 1.3 mV for the 2.5 μg/mL concentration; and 115 ± 43.9 and -33.95 ± 0.64 mV for the 10 μg/mL concentration. Even though aggregation was observed, we did not interfere with this natural phenomenon by adding surfactants as these could confound toxicity studies [41]. In addition, NPs are found in nature in both aggregated and unaggregated states and its important to evaluate effects of aggregated particles [42, 43]. Future studies should study aggregation patterns in test media as these could affect results [44].

### 3.1 Effects of PS NPs on gene expression of human neural stem cell line (hNS1)

To assess the molecular mechanisms of action of PS NPs, the expression changes of several genes in hNS1 cells were evaluated after PS NP exposure to three concentrations (0.5, 2.5 and 10 μg/mL) for 4 days. In general, we have found that NPs affected most gene expression in a concentration dependent manner, although some genes were upregulated while others were downregulated. Several genes were not altered.

### 3.2 Stress response: *hsp27/ hspB1*, *hsp90α*, and *hsp70/hspA5*

**Fig 1:**
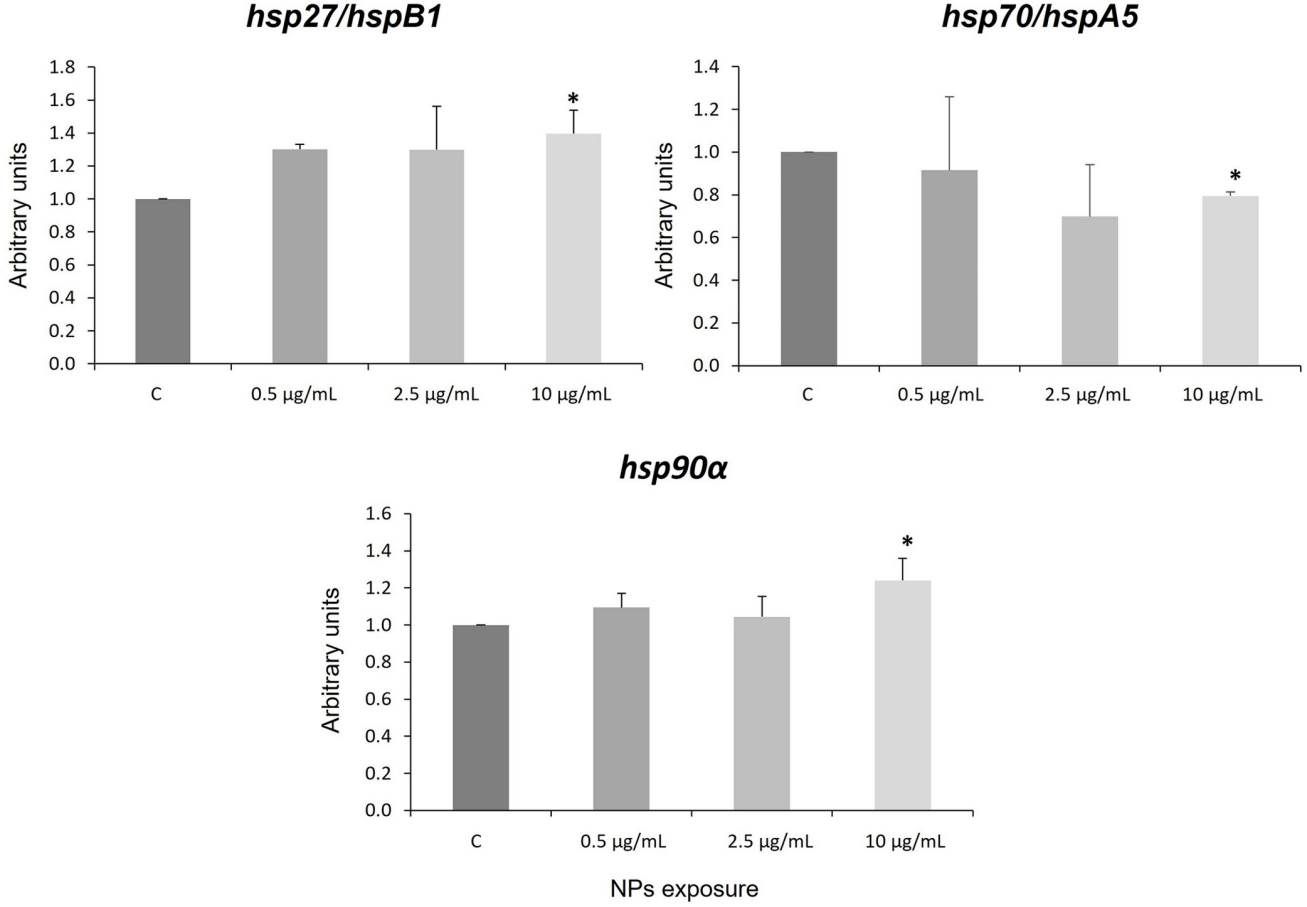
Expression levels of the *hsp27/hspB1*, *hsp70/hspA5*, and *hsp90*α genes. Statistical differences compared to control were marked with asterisks (*p*—value< 0.05). C: control. Error bars are based on the standard deviation.

HSPs belong to a large family of conserved proteins whose function is to maintain cellular balance in response to different external factors [45–47]. In this study, the alteration of three stress response genes (*hsp27/hspB1*, *hsp70/hspA5* and *hsp90α*) were assessed after 4 days of exposure. The results showed an increase of *hsp27/hspB1* and *hsp90α* mRNA expression by PS NPs at 0.5, 2.5 and 10 μg/mL (Fig 1), being statistically significant at the highest concentration.

In contrast, inhibition of *hsp70/hspA5* mRNA expression by PS NPs was observed at all concentrations studied, although only significant at 10 μg/mL. These results demonstrate that PS NPs alter the cellular response to stress in human neural cells. HSP27/HSPB1 belongs to the family of small heat shock protein (SHSPs, between 12 and 43 kDa) [48] which plays an essential physiological and pathophysiological role in diverse neurodegenerative diseases [47]. Members of this family, including HSP27/HSPB1, are expressed in the central nervous system in stress and non-stress situations [49] and are related to neurodegenerative diseases (Alzheimer’s, Alexander’s disease, multiple sclerosis) [47]. Moreover, The SHSPs interact with protein aggregates that present a detrimental conformation, such as β-amyloid peptide aggregates in Alzheimer’s disease, superoxide dismutase 1 in sclerosis, etc. Our data show that the presence of NPs can induce activation of *hsp27/hspB1* gene expression. This is important because previous studies show that it is upregulated in the brains of people with Alexander and Alzheimer’s diseases [50–52]. Moreover, HSP27/HSPB1 regulates the activation of proinflammatory genes [53] and the release of proinflammatory mediators [54], in reaction to cell damage or stress [55]. Furthermore, previous studies showed that when this protein is administered extracellularly it modulates immune and inflammatory processes [56], and it has also been shown to block the apoptotic pathway [57, 58]. On the other hand, the other two HSPs studied, HSP70/HSPA5 and HSP90α, are large ATP-dependent chaperones with a molecular mass of approximately 40 to 105 kDa. As mentioned above, our results seem to indicate that NPs modify the expression of both genes. *hsp70/hspA5* and *hsp90α* can be induced under stress, unlike the *hsp*90β isoform that is constitutively expressed [46]. In the presence of PS NPs, *hsp90α* expression levels increased. In contrast, treatment with PS NPs downregulated *hsp70/hspA5* expression. Inhibition of the antiapoptotic gene *hsp70/hspA5* has been described in Zfe [21], in human cells [26], and in *Apostichopus japonicus* [59] exposed to NPs. It appears that these nanomaterials alter the stress and anti-apoptotic response of the *hsp70/hspA5* gene. Previous studies suggest that this down-regulation of the *hsp70/hspA5* antiapoptotic gene may be related to entry into apoptosis [60, 61]. On the other hand, HSP70/HSPA5 has an alternate role in protein folding, together with HSP90α [62]. This is the first evidence that NPs induce *hsp90α* overexpression in human neuronal cells. However, *hsp70/hspA5* and *hsp90α* up-regulation has been observed in *Daphnia pulex* after treatment to 75 nm PS particles [63]. It is possible that in the face of the inhibition of *hsp70/hspA5* expression shown in our results, the correct folding carried out by HSP70/HSPA5 together with HSP90α [62] cannot take place and this has neurotoxic consequences in the cell by accumulation of misfolded proteins. Clearly further work is needed to corroborate these hypotheses.

### 3.3 Oxidative stress response: *Cu/Zn SOD1*, *MnSOD2*, and *cat*

**Fig 2:**
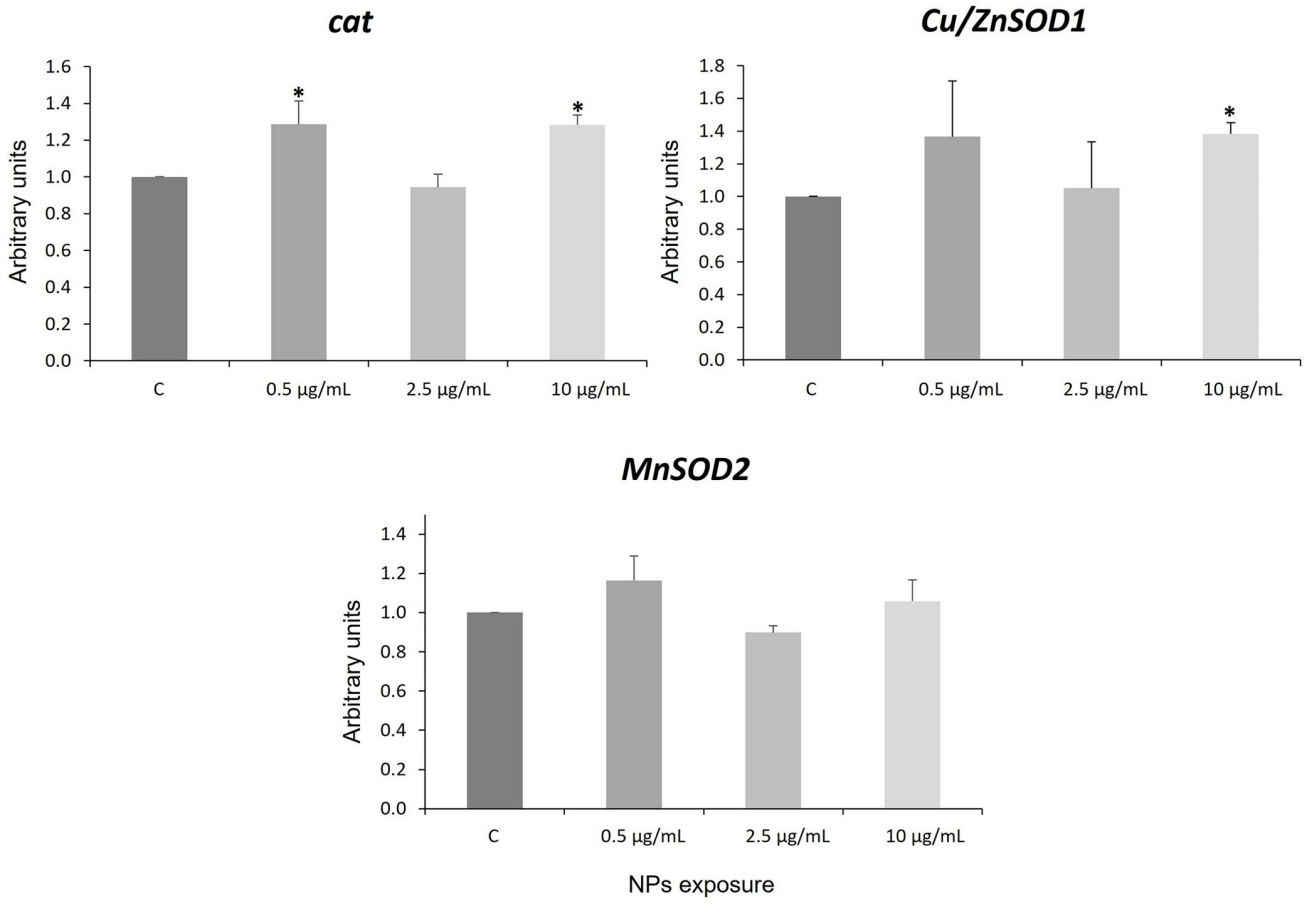
*Cat*, *Cu/ZnSOD1* and *MnSOD2* genes expression. Statistical differences of controls versus larvae exposed to NPs are indicated by asterisks (p-value < 0.05). C: control. Error bars are based on the standard deviation.

Previous studies have described that NPs can stimulate reactive oxygen species (ROS) production and cause oxidative stress in human cells [64], Zfe [17, 21, 63–65], and in *Daphnia pulex* and *Karenia mikimotoi* [66]. Increased ROS can generate oxidative stress, mitochondrial damage, amplified inflammatory cytokines and proapoptotic factors that could induce apoptosis in cells and in animals [56, 67–70]. Superoxide dismutase (SOD) and catalase (CAT) enzymes can be activated in the presence of ROS. These antioxidant enzymes have the function of protecting against ROS [71]. There are two different SOD isoenzymes (SOD1 and SOD2) present inside cells [72]. SOD 1 (Cu/ZnSOD1) is located in the cytoplasm and in the mitochondrial intermembrane space, peroxisomes, and nucleus. SOD 2 (MnSOD2) is a mitochondrial manganese protein, responsible for the removal of superoxide anions produced during oxidative phosphorylation. Catalase (CAT) is an enzyme that mitigates the toxic effects of hydrogen peroxide [73]. In this study, the effect of PS NPs (30 nm) on hNS1 induced changes in the expression of *Cu*/Zn*SOD1* and *cat* (Fig 2), specifically, it resulted in the activation of RNA expression of these oxidative stress-related genes, although not in a dose-dependent manner (Fig 2).

These results agree with data obtained in human HepG2 cells exposed to 50 nm NPs [74] and in zebrafish embryos (Zfe) exposed to 30 nm NPs [21]. However, the opposite result was reported by Aliakbarzadeh [30] who demonstrated that exposure to NPs inhibited CAT activity in zebrafish larvae. Oxidative stress can be considered as one of the molecular initiating events responsible for the toxicity of NPs [75].

### 3.4 DNA Damage response: *gadd45α*, *rad51*, *and xrcc1*

**Fig 3:**
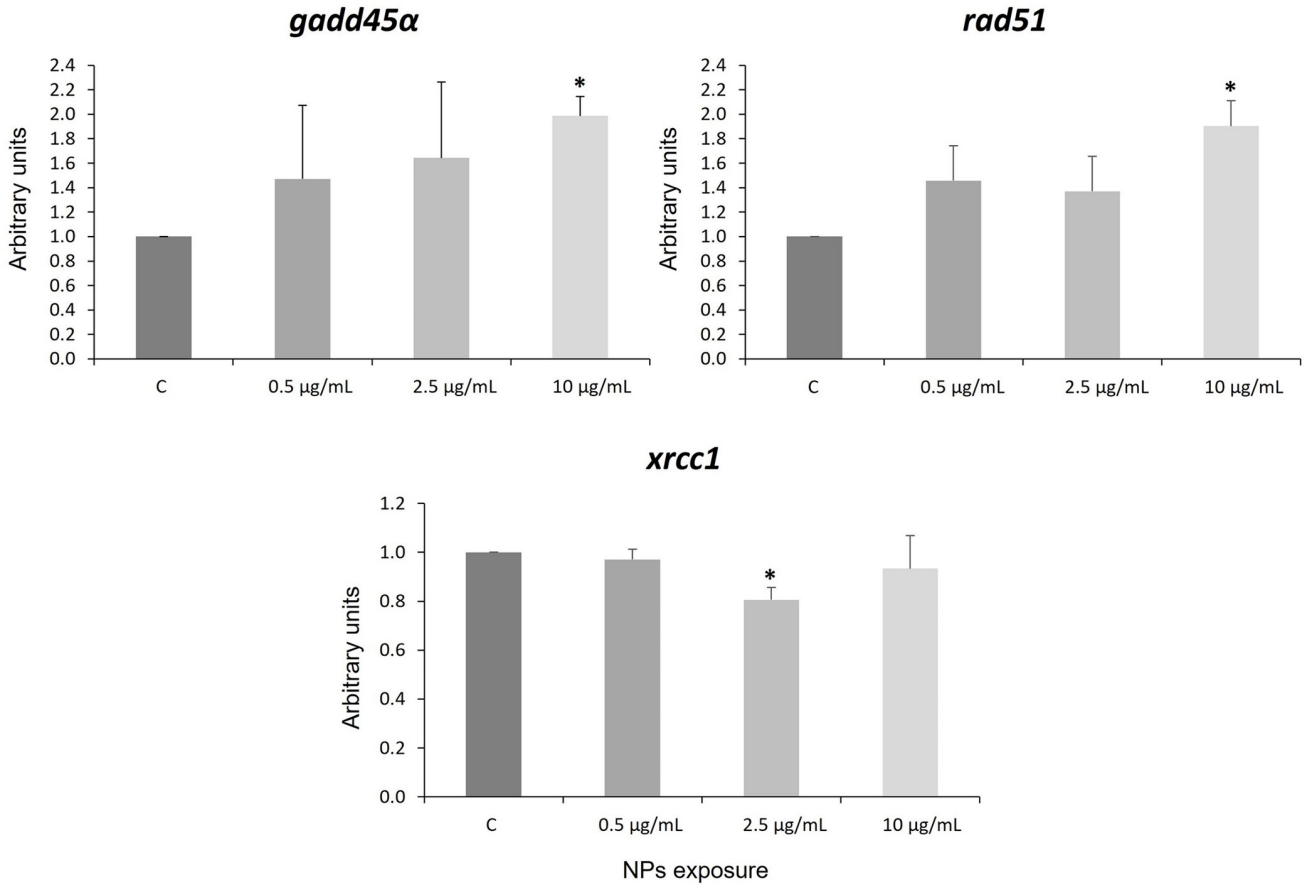
Representation of *gadd45*α, *rad51*, and *xrcc1* gene expression. Asterisks indicate statistical differences compared to controls (*p*-value < 0.05). C: control. Error bars are based on the standard deviation.

NPs can cause DNA damage in blood cells in mussels [76], in fish [77, 78], and in other aquatic species [79–82]. Most of these evaluations have been performed using the comet assay, but there are almost no studies that analyze the response at the molecular level of genes implicated in response to DNA damage. In this study, we decided to analyze the expression of three genes related to the DNA repair process, *gadd45α*, *rad51*, and *xrcc1* in human neural stem cells exposed to PS NPs for 4 days. Gadd45α is involved in the repair of DNA by nucleotide excision [83] and in mediating apoptosis induced by stress and genotoxic agents, such as pollutants or radiation. RAD51 is a protein implicated in DNA repair of double-strand breaks (DSBR), and their dysfunction is linked to diseases like cancer [84]. XRCC1 is a protein that interacts with multiple enzymatic components in single-stranded DNA break repair (SSBR) [85]. Together they are capable of accelerating SSRP and DSBR. In NP-treated hNS1 cells, the expression levels of the genes, *gadd45α* and *rad51*, were increased at the highest dose, whereas the *xrcc1* expression was decreased after NPs exposure, only significant at 2.5 μg/mL (Fig 3).

These results suggest that oxidative stress induced by NPs [26, 69, 86–88] can produce DNA damage [89]. Previous studies in mice with the *xrcc1* gene deletion show a link between DNA strand break repair and neurogenesis [90]. Also, deletion of *xrcc1* in the brain results in neuropathologies [90]. Inherited mutations in *xrcc1* lead to neurodevelopmental disorders and/or neurodegeneration [91, 92]. The inhibition of *xrcc1* expression and *hsp27/hspB1* activation in the presence of NPs suggests that these nanomaterials could cause DNA damage with neuropathological implications that could be related to neurodegenerative disorders. This information opens an avenue for further study of the possible damage of NPs at the level of the nervous system. Overexpression of *gadd45α* and *rad51* genes would indicate activation of DNA repair mechanisms by the presence of NPs, suggesting that DNA damage was likely caused by activation of oxidative stress induced in hNSCs. These results agree with those obtained in *Allium cepa* cells [93] but are in discordance with results obtained with NPs in Zfe [21]. The model used for the toxicity tests (whole organism *vs* cells) and the exposure conditions could explain the difference between the results. This is the first study demonstrating that NPs produce DNA damage in human neuronal cells.

### 3.5 Apoptotic response: *Cas3a*, *Cas7*, *p53* and anti-apoptotic gene *Bcl2*

**Fig 4:**
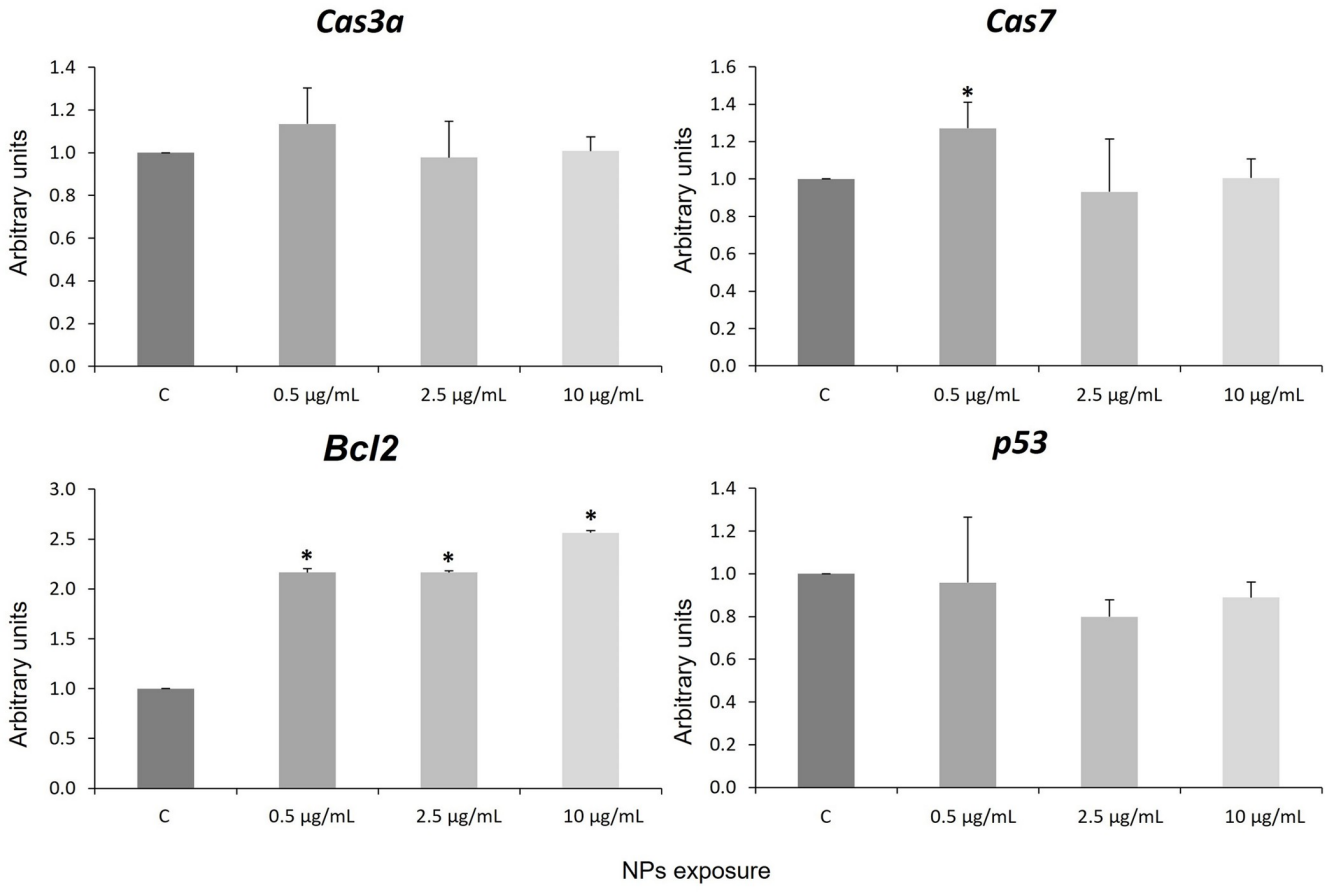
Expression of the *Cas3a*, *Cas7*, *Bcl2* and *p53* genes. Error bars are based on the standard deviation. Asterisks indicate statistical differences compared to controls (*p*-value < 0.05). C: control.

Quantitative polymerase chain reaction (qPCR) assays indicated that the mRNA level of *Cas7* increased significantly in cells treated with NPs at the lowest concentration, whereas *Bcl2* increased significantly at all concentrations studied and *Cas3α* and *p53* did not change expression levels (Fig 4). *Bcl2* is a member of the family of anti-apoptotic genes inhibiting proapoptotic genes [88]. The up regulation of this antiapoptotic gene could be a consequence of the entry into apoptosis and could be in agreement with the observed activation of the expression of the *hsp27/hspB1* gene, whose anti-apoptotic function has been described [57, 58]. This would suggest that NPs induce the expression of antiapoptotic proteins. This result agrees with those obtained in Zfe, *Crassostrea virginica*, and *Sterechinus neumayeri* exposed to NPs [94–96]. In contrast, our previous results showed down-regulation of *Bcl2* activity after NPs exposure in Zfe [21]. This discrepancy in results may be due to the difference in response to PS NPs from a whole organism or from a specific cell culture of hNSC, as in the Zfe model observe the response of all cell types within the individual, and not only neural cells. On the other hand, the exposure times were different in both models. Caspases are a family of highly conserved intracellular proteases cysteine-dependent intracellular proteases with an important role in inflammatory responses and apoptosis [92]. Caspases -3, -6 and -7 belong to the group of apoptosis executioners [97]. In this study, we analyzed the expression of two caspases (*Cas3a* and *Cas7*) involved in apoptosis. The results show a tendency to activate *Cas7* gene expression at the lowest concentration, but *Cas3a* was not significantly altered (Fig 4).

It is possible that predicted NP-induced apoptosis could be partially blocked by overexpression of anti-apoptotic genes (*Bcl2* and *hsp27/hspB1*). This is supported by previous results suggesting that the NPs activate programmed cell death in human lung epithelial cells [25]. Assays with shorter exposure times would probably allow us to test this hypothesis. The p53 tumor suppressor is a homotetrameric transcription factor involved in the control of cell proliferation and cell cycle, senescence, cell survival and apoptosis [88, 98]. ROS act as a key initiator in several signalling pathways involving cell cycle and energy metabolism [99]. Qiang and Cheng [100] have reported ROS-mediated activation of the p53 apoptotic cascade due to MPs exposure. Moreover, the *p53* gene transduces signals to stimulate apoptosis through activation of cas3b and gadd45ba [101, 102]. Our results show that *p53* gene expression does not change in human neuronal cells after exposure to NPs for 4 days (Fig 4).

In general, we hypothesize that the increased expression of the anti-apoptotic genes *Bcl2* and *hsp27/hspB1* after exposure of neuronal cells to NPs is possibly the result of activation of apoptosis as this process usually occurs as a consequence of oxidative stress and DNA damage induced by NPs. Ultimately, our results suggest that DNA damage and oxidative stress produced by NPs in human neuronal cells induce apoptosis. This is supported by the overexpression of the anti-apoptotic genes *Bcl2* and *hsp27/hspB1*, together with the activation of the apoptotic gene *Cas7*. Our results show that the executing caspases are only activated at the lowest concentration of NPs. It is possible that the anti-apoptotic response is preventing the cell from entering apoptosis.

### 3.6 Inflammatory and mitochondrial response: *IL-6*, *IL-8*, *TNF* and *Cox5A*

**Fig 5:**
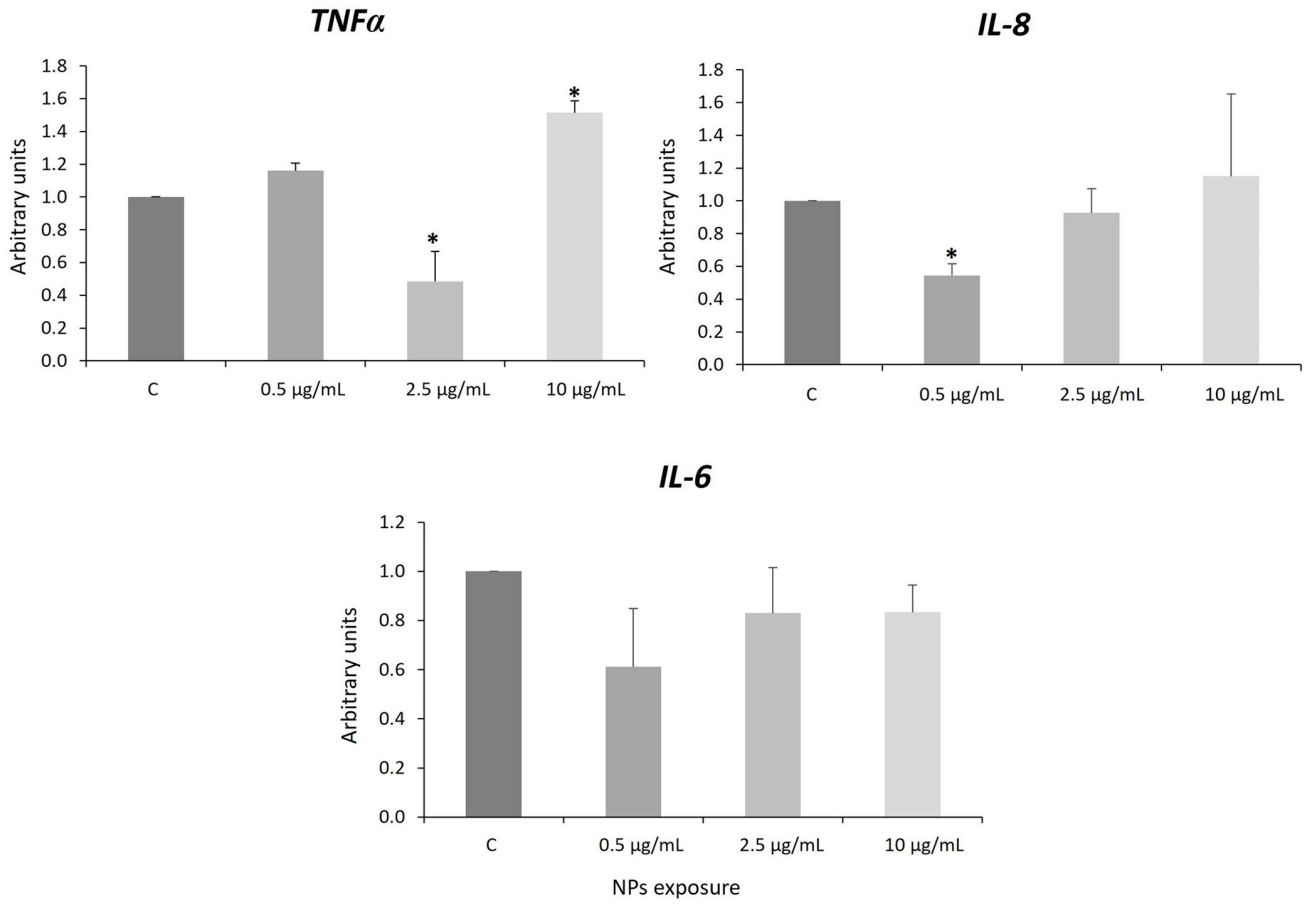
Expression of the *TNF*α, IL-8, and *IL-6* genes. Error bars are based on the standard deviation. Asterisks indicate statistical differences compared to controls (*p*-value < 0.05). C: control.

Our results show that PS NPs induced biphasic responses in these genes as they inhibited *TNFa* expression after 2.5 μg/mL exposure and activated *TNFa* expression after 10 μg/mL exposure (Fig 5).

Moreover, they inhibited the expression of *IL-6* at all concentrations used but specially at 0.5 μg/mL (albeit not significantly) and *IL-8* gene when cells are exposed to 0.5 μg/mL (Fig 5). However, as the graph shows (Fig 5), a trend of *IL-8* gene activation is observed when we increased the concentration of PS NPs. Therefore, here we demonstrate that PS NPs can modify the expression of *TNFα* and *IL-6* and *IL-8*, which are essential genetic markers in the inflammatory mechanisms. Activation of the inflammatory response has been described in human lung epithelial cells [25] and in Zfe exposed to NPs [14, 21, 103]. On the other hand, it has been previously shown in human synovial fibroblasts (SFC) that proinflammatory cytokines such as TNF, IL-1, and IL-6 can induce the expression of *hsp70/hspA5* under cellular stress [104] in order to protect the cell from apoptosis. It is possible that the inhibition of *hsp70/hspA5* observed in our results after exposure to PS NPs is a consequence of the non-activation of one of the proinflammatory genes analyzed (*IL-6*). Our results suggest that NPs can modify the inflammatory response in human neural stem cells, but this depended on the concentration of the NPs.

**Fig 6:**
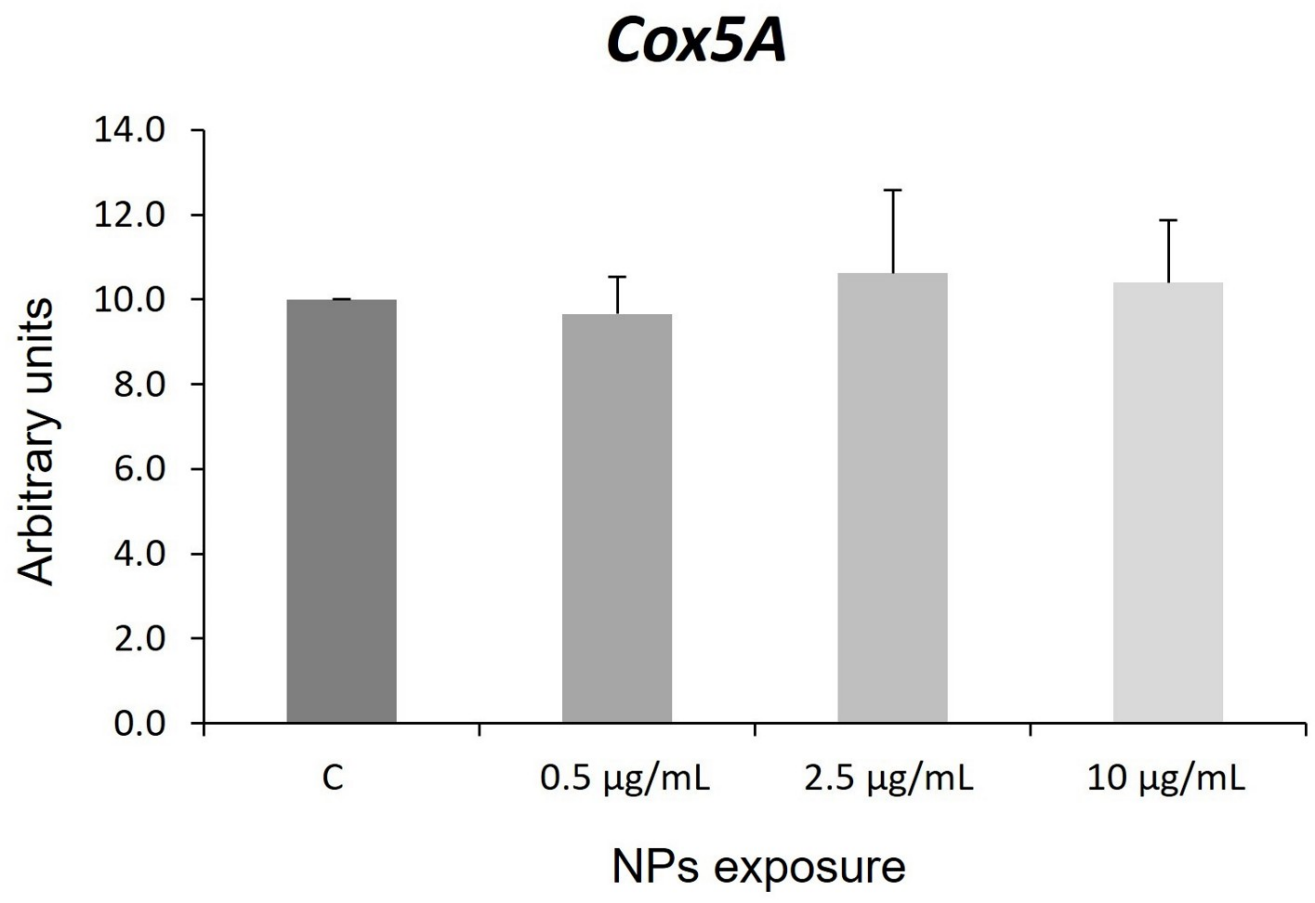
Expression of the *Cox5A* gene. C: control. Error bars are based on the standard deviation.

Cytochrome c oxidase (COX) is a mitochondrial enzyme of the respiratory chain. This protein is implicated in proton pumping and is essential for ATP synthesis. Deficient COX5A expression significantly impairs COX function, thus causing mitochondrial dysfunction in skeletal muscle, pulmonary arterial hypertension, and growth retardation [105, 106]. COX has a regulatory role in electron transport [107]. Many xenobiotics inhibit COX activity and generate mitochondrial stress, but their mechanisms are unknown [108, 109]. However, little is known about the role of COX5A in the response to pollutants. Our results showed that hNS1 cells exposed to different concentrations of NPs did not modify *Cox5A* expression (Fig 6).

These results contradict previous data and possible differences in NP size and/or concentrations could be responsible for these differences [110, 111].

## 4. Conclusions

Our results show that *Cu/ZnSOD 1* and *cat* expression are activated in hNSC exposed to PS NPs. This antioxidant response may be a consequence of the production of reactive oxygen species (ROS) induced by NPs entering the cells. Possibly the antioxidant metabolism response is not sufficient to stop ROS damage to DNA. As a consequence of this DNA damage, the expression levels of genes involved in DNA repair (*gadd45a* and *rad51*) are increased, although an inhibition of *xrcc1* is also seen. All this suggests that DNA repair mechanisms are altered in hNSC treated with PS NPs. The oxidative stress and DNA damage produced by NPs would activate cell apoptosis. This is supported by the increased expression of anti-apoptotic genes, such as *Bcl2* and *hsp27/hspB1*, together with the apoptotic gene *Cas7*. However, the up regulation of anti-apoptotic genes (*Bcl2* and *hsp27/hspB1*) would not have been sufficient to block the activation of apoptotic gene expression (*Cas7*). It is possible that there is a partial blockade of these apoptotic genes at the concentrations and times studied, but further research is needed in this line of investigation. On the other hand, inhibition of *hsp70*/*hspA5*, a chaperone involved in the early steps of protein folding, would avoid the activity of HSP90α. This would lead to the accumulation of misfolded proteins and potential cellular neurotoxic effects. Finally, NPs alter the inflammatory response (*TNFα*, *IL-6 and IL-8)* of hNSC, but effects depended on concentrations, and possibly timing and size of NPs studied. The results of this study suggest that PS NPs can cause damage and functional alterations in human neuronal cells. Therefore, the effects of NPs on pathways related to neurodevelopmental problems and neurodegenerative diseases need to be further investigated.
